# Supplementary material for: ProteinShader: illustrative rendering of macromolecules
Source: BMC Struct Biol. 2009 Mar 30;9:19. doi: 10.1186/1472-6807-9-19 (PMC2672931; doi:10.1186/1472-6807-9-19)
Supplement: Additional file 1 — ProteinShader program without source code. This compressed file contains the complete ProteinShader program including associated libraries, but no source code. A README.txt file gives an overview of the ProteinShader distribution, and the index.html file in the help subdirectory has directions on getting started with the program as well as a set of tutorials. [file 1472-6807-9-19-S1.zip › ProteinShader-beta-0_9_4-binary/help/api/org/proteinshader/gui/listeners/controlpanel/AntialiasingPanelListenerFactory.html]

AntialiasingPanelListenerFactory (ProteinShader API)


|  |  |  |
| --- | --- | --- |
| **Overview** \| **Package** \| **Class** \| **Use** \| **Tree** \| **Deprecated** \| **Index** \| **Help** | |  |
| PREV CLASS   **NEXT CLASS** | **FRAMES**    **NO FRAMES**     **All Classes** |  |
| SUMMARY: NESTED \| FIELD \| CONSTR \| METHOD | DETAIL: FIELD \| CONSTR \| METHOD |  |


---


## org.proteinshader.gui.listeners.controlpanel Class AntialiasingPanelListenerFactory

```
java.lang.Object
  org.proteinshader.gui.listeners.controlpanel.AntialiasingPanelListenerFactory
```

---

``` public class AntialiasingPanelListenerFactory extends Object ```

Creates the listeners for the AntialiasingPanel.

---

**Constructor Summary**

`AntialiasingPanelListenerFactory(Mediator mediator,
AntialiasingPanel antialiasingPanel)`
  
          Constructs a AntialiasingPanelListenerFactory.


| **Method Summary** | |
| --- | --- |
| `ItemListener` | `createHalftoningCheckBoxListener()`             Creates a change listener for the 'Antialias halftoning images' check box. |
| `ActionListener` | `createJitterMenuActionListener()`             Creates a listener for the Jitter menu. |
| `ItemListener` | `createSceneCheckBoxListener()`             Creates a change listener for the 'Antialias entire scene' check box. |

**Methods inherited from class java.lang.Object**

`clone, equals, finalize, getClass, hashCode, notify, notifyAll, toString, wait, wait, wait`

**Constructor Detail**

### AntialiasingPanelListenerFactory

```
public AntialiasingPanelListenerFactory(Mediator mediator,
                                        AntialiasingPanel antialiasingPanel)
```

:   Constructs a AntialiasingPanelListenerFactory.

    **Parameters:**: `mediator` - the centralized Mediator object that listeners call on to accomplish their task.: `antialiasingPanel` - the AntialiasingPanel that this class creates listeners for.


**Method Detail**

### createSceneCheckBoxListener

```
public ItemListener createSceneCheckBoxListener()
```

:   Creates a change listener for the 'Antialias entire scene' check box.

---


### createHalftoningCheckBoxListener

```
public ItemListener createHalftoningCheckBoxListener()
```

:   Creates a change listener for the 'Antialias halftoning images' check box.

---


### createJitterMenuActionListener

```
public ActionListener createJitterMenuActionListener()
```

:   Creates a listener for the Jitter menu.


---


|  |  |  |
| --- | --- | --- |
| **Overview** \| **Package** \| **Class** \| **Use** \| **Tree** \| **Deprecated** \| **Index** \| **Help** | |  |
| PREV CLASS   **NEXT CLASS** | **FRAMES**    **NO FRAMES**     **All Classes** |  |
| SUMMARY: NESTED \| FIELD \| CONSTR \| METHOD | DETAIL: FIELD \| CONSTR \| METHOD |  |


---

# *Copyright © 2007-2008*
